# The sensitivity of the DNA damage checkpoint prevents oocyte maturation in endometriosis

**DOI:** 10.1038/srep36994

**Published:** 2016-11-14

**Authors:** Mukhri Hamdan, Keith T. Jones, Ying Cheong, Simon I. R. Lane

**Affiliations:** 1 Human Development and Health Academic Unit, University of Southampton, Faculty of Medicine, and Complete Fertility Centre Southampton, Princess Anne Hospital, Southampton, UK; 2 Department of Obstetrics and Gynecology, Faculty of Medicine, University of Malaya, 59100 Kuala Lumpur, Malaysia; 3 Centre for Biological Sciences, Faculty of Natural and Environmental Sciences, University of Southampton, Southampton SO17 1BJ, UK

## Abstract

Mouse oocytes respond to DNA damage by arresting in meiosis I through activity of the Spindle Assembly Checkpoint (SAC) and DNA Damage Response (DDR) pathways. It is currently not known if DNA damage is the primary trigger for arrest, or if the pathway is sensitive to levels of DNA damage experienced physiologically. Here, using follicular fluid from patients with the disease endometriosis, which affects 10% of women and is associated with reduced fertility, we find raised levels of Reactive Oxygen Species (ROS), which generate DNA damage and turn on the DDR-SAC pathway. Only follicular fluid from patients with endometriosis, and not controls, produced ROS and damaged DNA in the oocyte. This activated ATM kinase, leading to SAC mediated metaphase I arrest. Completion of meiosis I could be restored by ROS scavengers, showing this is the primary trigger for arrest and offering a novel clinical therapeutic treatment. This study establishes a clinical relevance to the DDR induced SAC in oocytes. It helps explain how oocytes respond to a highly prevalent human disease and the reduced fertility associated with endometriosis.

There is an established correlation between endometriosis and reduced success in spontaneous or medically assisted conception^1^,^2^,^3^. Additionally, evidence that reduced fertility in endometriosis is exerted through the oocyte, and not other factors is accumulating^4^. For instance, implantation and pregnancy rate are not significantly different in patients with endometriosis compared to controls when undergoing IVF and embryo transfer^5^,^6^. Further evidence comes from oocyte donor studies: control donor oocytes were equally likely to implant in patients with endometriosis as without^7^,^8^, whilst oocytes from women with endometriosis were less likely to implant than those without^9^. Together these data suggest that the reduced clinical pregnancy rates are likely due to oocyte quality.

Development of mature, fertilizable eggs from immature ‘germinal vesicle’ (GV) oocytes takes place in antral follicles prior to ovulation. The checkpoint that controls completion of oocyte maturation is the Spindle Assembly Checkpoint (SAC), named after its ability to prevent progression through the cell cycle until the spindle fully formed and chromosomes attached correctly in preparation for anaphase. We and others have recently shown an additional function of this checkpoint, unique to oocytes: its ability to prevent maturation in response to DNA damage^10^,^11^,^12^,^13^. As yet it is untested whether the extent of DNA damage required to activate the SAC-DDR checkpoint can occur pathologically or even physiologically.

Endometriosis is a disease associated with elevated ROS, both in the follicular environment and systemically^14^,^15^,^16^,^17^,^18^. It is also known that ROS causes DNA damage^19^. Thus we reasoned that ROS associated with endometriosis may cause DNA damage in oocytes, and impair oocyte maturation, potentially accounting for reduced fertility in the disease. To test this hypothesis we use follicular fluid from patients with endometriosis to investigate the effects of physiological levels of ROS and DNA damage on oocyte maturation.

## Results

### Follicular Fluid from patients with endometriosis reduces mouse oocyte maturation

**Figure 1:**
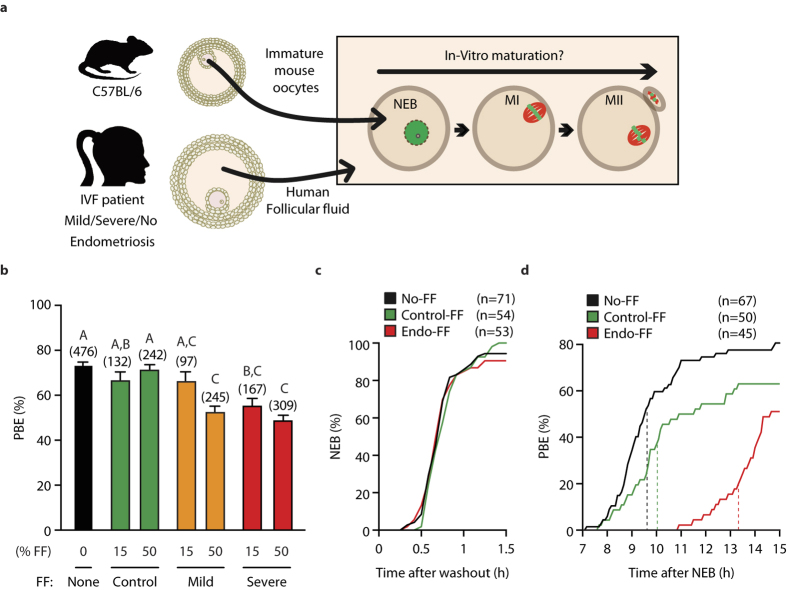
Incubation in follicular fluid from patients with endometriosis delays or prevents oocyte maturation in mouse. (**a**) Mouse oocytes were incubated in media containing human follicular fluid and later observed for Nuclear Envelope Breakdown (NEB) and Polar Body Extrusion (PBE). (**b**) Rate of PBE following 16 h incubation in media containing follicular fluid. Groups without common letters indicate statistical difference (Fisher’s exact test with Bonferroni correction, P < 0.05). Number of oocytes are shown in parenthesis, bars represent standard error. Experiments were repeated 7–10 times using follicular fluid from 3–7 different patients. (**c**,**d**) Following timelapse imaging of mouse oocytes in follicular fluid the time of NEB (**c**) and PBE (**d**) were determined and plotted cumulatively. Dashed vertical lines indicate the mean timing of PBE.

Immature oocytes harvested from young C57/BL6 mice were cultured in follicular fluid from patients undergoing IVF who were laparoscopically confirmed as having mild, severe or no endometriosis (ASMR classification^20^, Fig. 1a). Patient groups showed no significant differences in key fertility measures, except with endometriosis fewer oocytes were retrieved (Table S1). Oocytes were assessed for the formation of a polar body, which marks the conclusion of Meiosis I, at 14–16 hours after washout from milrinone. This time period encompasses the physiologically relevant window for mouse oocyte maturation. Oocytes cultured with either 15% or 50% follicular fluid from control patients showed no difference in their maturation rate, measured by polar body extrusion (PBE), compared to those cultured without follicular fluid (P = 0.1563, P = 0.5963 respectively; Fig. 1b). Similarly 15% FF from mild endometriosis patients had no significant impact on maturation (P = 0.1728). However, 50% follicular fluid did, reducing PBE rates from 73% (n = 476) to 52% (n = 245; P < 0.0001). In the severe endometriosis groups both 15% and 50% follicular fluid were found to significantly reduce PBE (55%, n = 167; 49%, n = 309; respectively P < 0.0001). Since no statistical difference between mild and severe follicular fluid was found (Fig. 1b; individual patients, Figure S1), subsequent experiments used only 50% severe follicular fluid, termed ‘Endo-FF’ from individual patients.

To further examine the reduced ability to complete meiosis I in the Endo-FF group we used timelapse microscopy. We found that neither Control-Follicular Fluid (Control-FF) nor Endo-FF had any impact on the timing of NEB (Fig. 1c). However, there was a pronounced effect on the timing of PBE, with Endo-FF causing a delay of over 3 h (Fig. 1d, dashed lines; 13.3 h vs 10.0 h; P < 0.0001). Together, these observations suggest Endo-FF has an inhibitory effect on oocyte maturation, leading to a meiotic arrest or delay.

### Follicular fluid from patients with endometriosis generates ROS and DNA damage in oocytes

**Figure 2:**
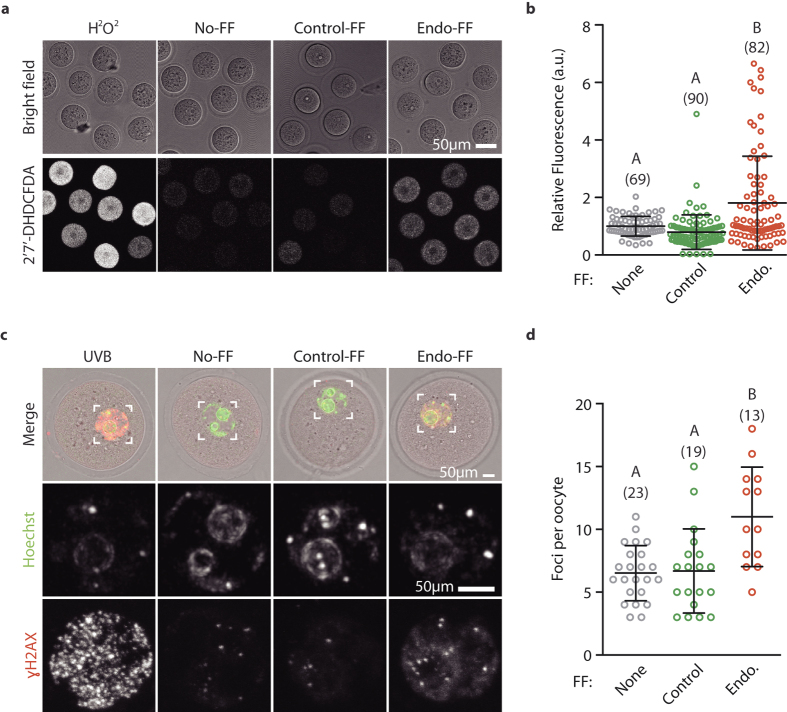
Endometriosis follicular fluid generates reactive oxygen species and causes DNA damage in oocytes. (**a**) Representative brightfield and fluorescence images of oocytes loaded with the ROS indicator 2′7′-DHDCFDA and exposed to H_2_O_2_, Control-FF, Endo-FF or No-FF. Scale bar represents 50 μm. (**b**) Fluorescence readings from oocytes shown in (**a**) Normalised to No-FF group. Number of oocytes shown in parenthesis, data from 3 independent experiments. (**c**) Representative images showing merged (top) chromatin (middle) and γH2AX (bottom) staining in oocytes. The nuclear region (top row, white box) is enlarged in the Hoechst and γH2AX images. Oocytes treated with UV light (positive control) or incubated in, Control-FF, Endo-FF or No-FF as indicated. (**d**) Number of γH2AX foci per oocyte from (**c**). (**b**,**d**) Groups without common letters indicate statistical difference (**b)**, P < 0.001; (**d**), P < 0.05; ANOVA with Tukey’s post-hoc test).

Next we tested follicular fluid for its ability to raise levels of ROS in oocytes. We pre-incubated mouse oocytes with a fluorescein based ROS reporter^21^ then exposed them to media containing H_2_O_2_, No-FF, Control-FF or Endo-FF (Fig. 2a). H_2_O_2,_ which generates high levels of ROS, was used as a positive control. Importantly Endo-FF caused significantly higher ROS production when compared to either Control-FF or No-FF (1.81 ± 1.63 vs 1.00 ± 0.34 or 0.80 ± 0.60, P < 0.0001; Fig. 2b).

ROS is a known source of DNA damage^19^, we therefore tested the extent of DNA damage present in oocytes by examining histone H2AX phosphorylation (γH2AX), an event induced by DNA damage response kinases ATM, ATR or DNA-PK in the vicinity of damage sites^22^. Oocytes were incubated with Control-FF, Endo-FF, or No-FF or exposed to ultraviolet light (UV-B) as a positive control, and examined for γH2AX (Fig. 2c). As expected, UV-B resulted in a strong nuclear γH2AX signal. We found a significant rise in the mean number of γH2AX foci number in the Endo-FF group compared to No-FF or Control-FF (Endo-FF, 11.0 ± 4.0; Control-FF, 6.7 ± 3.4, P = 0.0008; No-FF, 6.5 ± 2.2, P = 0.0003; Fig. 2d). We note that whilst all oocytes treated with Endo-FF had raised levels of DNA damage, not all had raised levels of ROS, this may suggest that the γH2AX assay is more sensitive than the ROS indicator.

### Oocyte arrest is not due to spindle or chromosome attachment perturbations

**Figure 3:**
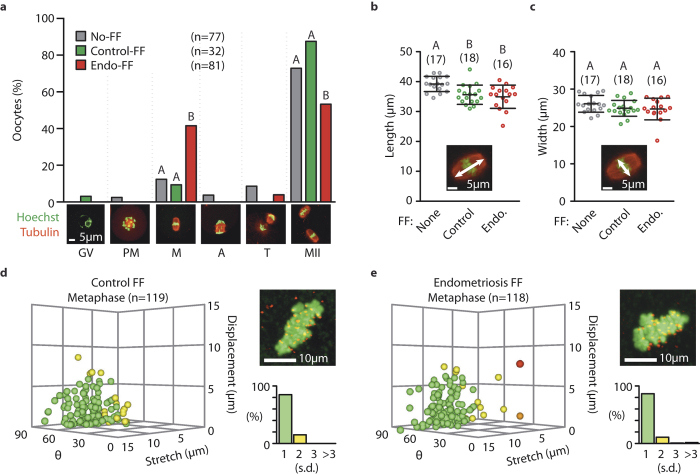
Endometriosis follicular fluid causes metaphase arrest, but does not disrupt spindles or metaphase chromosome alignment. (**a**) Oocytes fixed after 16 h incubation in follicular fluid were stained for tubulin and chromatin and then categorised morphologically for progression through meiosis (GV, germinal vesicle; PM, prometaphase; M, metaphase; A, anaphase; T, telophase; MII, metaphase II). Fisher’s exact test with Bonferroni multiple comparison correction was used to test significance. (**b**,**c**) Spindle dimensions of metaphase arrested oocytes in Endo-FF and time matched controls showing spindle length (**b**) and width (**c**). Number of oocytes are indicated in parenthesis. Groups without common letters indicate statistical difference (P < 0.05; ANOVA, Tukey’s post-hoc test). Error is standard deviation. Representative micrographs shows measurement (white arrow) with tubulin and chromatin (red and green respectively). (**d**,**e**) Scatter plots showing metaphase bivalent stretch, displacement and θ for each bivalent in the Control-FF (**d**) and Endo-FF (**e**) groups. Colour coding of points corresponds to the maximum number of standard deviations from the mean position in any axis, where the mean and s.d. are defined by No-FF metaphase oocytes (green, <1 s.d.; yellow, <2 s.d.; orange, <3 s.d.; red, ≥3 s.d.; see Figure S3 and methods for detail). Micrographs show representative images (Anti-centromeric Antibody, red; Hoechst, green). Bar charts show the percentage of bivalents according to the number of s.d. from the mean.

We next examined where in meiosis I oocytes were delayed/arrested. Following 16 h of culture, spindle morphology was examined by immunofluorescence. We found arrested oocytes were typically at metaphase I, consistent with a SAC arrest (Fig. 3a). In contrast with studies using follicular fluid in bovine oocytes^23^,^24^ no abnormal spindle morphology was found in any group. Further, when automated measurements were made of spindle length and width, these were indistinguishable between Endo-FF and Control-FF at metaphase I or II (Figs 3b,c and S2).

One potential reason for a metaphase arrest is a failure of bivalents to biorientate on the meiotic spindle, activating the SAC through the canonical route^25^. Bivalent biorientation can be measured indirectly because incorrectly attached chromosomes tend to be under less tension (stretch), be further away from the spindle equator (displacement) and tend not to be orientated along the long axis of the spindle (θ)^10^,^26^,^27^ (Figure S3A). No significant differences in these parameters were found between Control-FF and Endo-FF (Figs 3d,e and S3B–F). Therefore there is no evidence that the metaphase I arrest is caused by defects in the spindle, or by a failure of bivalents to establish biorientation.

### Oocyte maturation is rescued by inhibiting ROS, the DDR or the SAC

**Figure 4:**
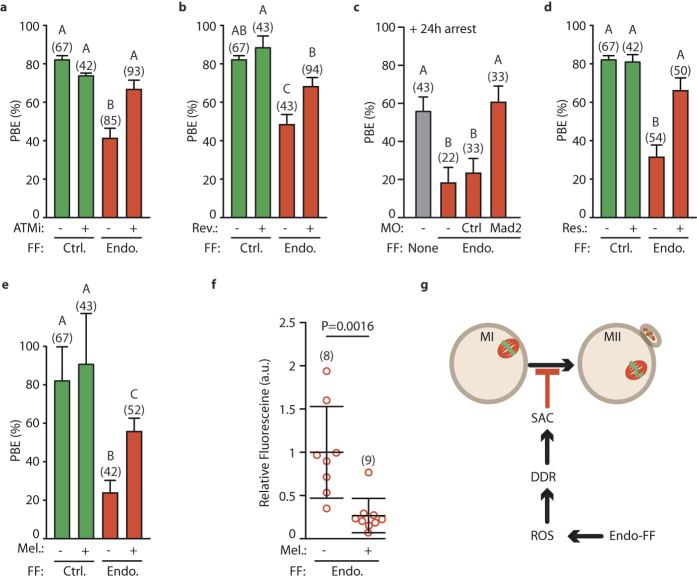
ROS produced by endometrial follicular fluid blocks oocyte maturation. (**a**) PBE rate following incubation in Control-FF or Endometriosis-FF with or without ATMi (40 μM). (**b**) PBE rate following incubation of oocytes in Control-FF or Endometriosis-FF with or without reversine (100 nM). (**c**) PBE rate after 24 hour culture following microinjection of morpholino against Mad2, a control morpholino, or no morpholino in the presence or absence of Endo-FF. (**d**,**e**) PBE rate of oocytes cultured in Control-FF or Endo-FF with or without addition of resveratrol (2 μM, d) or melatonin (100 μM, e).(**f**) Relative 2′7′-DHDCFDA fluorescence in oocytes incubated in Endo-FF with or without addition of melatonin (100 μM). (**a**–**f**) Number of oocytes shown in parenthesis, data from 2 or 3 independent experiments. Groups without common letters indicate statistical difference (P < 0.05). Error bars represent standard errors (**a**–**e**) or standard deviation (**f**). Statistical test used were Fisher’s exact test with Bonferroni correction (**a**–**e**), or unpaired t-test (**f**). (**g**) Proposed model showing the mechanism by which oocyte maturation is prevented or delayed in Endo-FF.

We next tested our hypothesis that the levels of DNA damage and ROS associated with Endo-FF would activate the recently identified DDR-SAC pathway^10^,^11^,^12^. To this end we inhibited ATM, an upstream kinase in the DDR pathway, with an ATM kinase inhibitor (ATMi)^28^,^29^. A significant 26% increase in PBE was seen in Endo-FF treated oocytes when co-incubated with ATMi (67% vs 41%, P = 0.0009; Fig. 4a), suggesting that ATM kinase activity is required for the arrest induced following exposure to Endo-FF.

Activation of the SAC in oocytes following DNA damage has been demonstrated previously^10^,^11^ providing a mechanism by which DNA damage would cause a metaphase I arrest. To test for SAC involvement oocytes were matured in Endo-FF and reversine, an Mps1 kinase inhibitor used previously to overcome the SAC^30^,^31^,^32^,^33^. Oocyte maturation was significantly increased by 20% following reversine addition (68% vs 48%, P = 0.0074; Fig. 4b). To further confirm the involvement of the SAC in Endo-FF arrest we reduced expression of Mad2, an integral member of the SAC, by antisense knockdown. In this experiment control oocytes had a PBE rate of 56%, lower than in other experiments, owing to the necessity to arrest oocytes for 24 h prior to maturation (Fig. 4c). Maturation in Endo-FF reduced this rate to 18%. The use of a control antisense morpholino (targeted to Mad2 5′ UTR but with 5 bases mis-matched^34^) did not significantly restore PBE rates (23%), however the Mad2 morpholino did (61%, P = 0.0047) to a rate indistinguishable from oocytes matured without Endo-FF. Together these data suggest Mad2 and Mps1, and therefore by implication the SAC, are essential for the metaphase I arrest or delay seen following exposure to Endo-FF.

Thus we have established that DNA damage present at levels achievable in disease can arrest mouse oocytes at metaphase I, by way of the DNA damage response and the SAC. To test if ROS was responsible for the MI arrest associated with Endo-FF, we utilised the antioxidants resveratrol and melatonin^35^,^36^,^37^. Oocytes matured in Endo-FF were >2-fold more likely to undergo PBE in the presence of either 2 μM resveratrol (P < 0.0001; Fig. 4d) or 25 ng/mL melatonin (P < 0.0001; Fig. 4e). The rescue of PBE was likely as a result of reduced ROS, as we could demonstrate a significant drop in ROS reporter fluorescence when oocytes were incubated with melatonin in the presence of Endo-FF (1.00 ± 0.53 vs 0.27 ± 0.20, P = 0.0016; Fig. 4f). These data suggest that ROS production is triggering the MI arrest caused by Endo-FF.

## Discussion

Our data implicate ROS, the DDR and the SAC in the metaphase I delay/arrest caused by follicular fluid from patients with endometriosis. Several lines of evidence support this: (i) Endo-FF raises ROS levels in oocytes, (ii) The DDR is switched on because γH2AX foci are formed, and ATM inhibition rescues PBE, (iii) The SAC is activated as shown by pharmacological Mps1 inhibition and Mad2 knockdown which both rescue PBE, further the SAC is not activated by poor spindle assembly or bivalent biorientation. It seems plausible that it is ROS causing DNA damage, so leading to the metaphase arrest through SAC activation. However, this will require future work as a direct link between ROS and DNA damage is not proven. It cannot be ruled out that an additional factor is present in follicular fluid that causes metaphase arrest independent of the DDR.

Importantly we show for the first time that the SAC pathway can be activated by brief exposure to dilute human follicular fluid from patients with endometriosis. In the human ovary, oocytes would be exposed to follicular fluid at higher concentrations and for longer periods of time. Therefore the pathway is likely highly sensitive to diseases such as endometriosis, and possibly others that could elevate ROS. Encouragingly, although the pathway is sensitive it can also be reversed *in-vitro* by anti-oxidant treatment. Reducing oxidative stress in the oocyte may therefore be of clinical importance when treating sub-fertility in endometriosis either *in-vivo* or *in-vitro*.

## Methods

### Study approval

We received approval from University Hospital Southampton and Hampshire B ethical committee. All procedures conducted in this study requiring human involvement were performed with informed consent and in accordance with ethical committee guidelines (Study on Human Oocyte, RHMO&G0213, REC13/SC/0551).

### Statistics

P values < 0.05 were considered significant. Fishers exact test (Dichotomous data) with correction for multiple comparisons where applicable or ANOVA with Tukey’s multiple comparison (continuous data) were performed using Prism software (GraphPad Software, Inc., USA). All chemicals and reagents were from Sigma Aldrich (UK) unless stated otherwise.

### Animals & oocyte culture

All experimental protocols were approved by the University of Southampton Animal Ethics Committee and carried out in accordance with UK Home Office regulations and the UK Animals (Scientific Procedures) Act of 1986 (ASPA) under UK Home Office licenses. Three-to-four-week old C57Bl6 female mice (Charles River, UK) were used. GV-stage oocytes were released from the ovaries of hormonally primed females 44–52 h following 10 IU PMSG-Intervet intraperitoneal injection (Centaur Services, UK). M2 medium supplemented with milrinone (1 mM) was used for collection and to maintain prophase arrest. Oocytes were mechanically stripped from the surrounding cells^38^. To determine maturation rates, GV oocytes were washed into fresh M2 or MEM (Amsbio, UK) media and checked for polar bodies 14–16 h later. When needed media was supplemented with ATMi (40 μM, Selleckchem, USA), reversine (100 nM), resveratrol (2 μM) or melotonin (25 μg/mL). All drugs were dissolved in dimethylsulphoxide (DMSO) or ethanol and used at dilutions of 0.1% or below. FF from an individual patient at 0, 15 or 50% was added to media, giving a total volume of 150 μL, and placed into wells of 96 well plates. Wells were covered by 30 μL embryo grade mineral oil to prevent evaporation. For timelapse imaging glass-bottom 96 well plates were used (P96G-0-5-F, MatTek, USA).

### Antibodies & Immunofluorescence

Oocytes were fixed (2% paraformaldehyde, 0.05% TritonX-100 in PHEM, 15 min), then permeabilised (0.05% TritonX-100 in PHEM, 30 min) at room temperature in a humidified chamber in preparation for immunostaining. Oocytes were washed three times in PBS-PVP and then blocked in 7% goat serum in PBS (1 h, room temperature). The following primary antibodies were used at 37 °C in blocking solution for 1 hour: rabbit anti-γH2AX, 1:200, (ab11174, Abcam, UK); anti-α tubulin, 1:400, (A11126, Life Technologies, UK); human anti-centromere antigen, 1:400, (90CCS1058, Bioclone, Australia). Following 5 washes, oocytes were then incubated in appropriate Alexa-conjugated secondary antibodies at 1:1000 dilutions for 1 h at room temperature. Following 5 more washes oocytes were stained with Hoechst (20 mg/mL) and mounted in Citifluor (Citifluor Ltd, UK).

### Microinjection and morpholino

Mad2 (5′-ATGGCACAGCAGCTCGCCCGAGAGC-3′) and Mad2 5-base mismatch (ATGGCGCTGCAGCTCTCCCGGGAGC) morpholinos (Gene Tools LLC, USA)^34^, were diluted in water, and introduced into oocytes by microinjection at tip concentrations of 1 mM. Microinjections into oocytes were performed on the stage of an inverted TE300 microscope (Nikon, Japan), at room temperature, using micromanipulators (Narishige, Japan). A single injection with a 0.1–0.3% volume was achieved using timed injection on a Pneumatic Picopump (World Precision Instruments, UK)^38^. Oocytes were incubated in MEM media with 5% CO_2_ for 24 h to allow for protein knockdown.

### Imaging

All images were acquired on a Leica SP8 confocal microscope fitted with hybrid detectors. For fixed specimens a 63x oil immersion lens and a z-resolution of 0.5 μm (ACA, γH2AX) or 2 μm (Tubulin) was used. Fluorchromes were imaged sequentially to avoid bleed-through. Live time-lapse imaging was performed with a 633 nm laser and images were acquired every 5 minutes using a 10x or 20x objective. The microscope had a 37 °C environmental chamber and lab-made software was used to perform time-lapse of multiple stage locations within one experiment.

### DNA damage quantification

Oocytes were exposed to follicular fluid for 1 hour before fixation and immunofluorescence. All groups were imaged using identical confocal settings on the same day. Following imaging of γH2AX, confocal stacks were cropped to leave only the nucleus and processed using the following ImageJ (NIH, USA) plugins as described previously^12^: Images were de-noised with ‘PureDenoise’ Plugin^39^. The ‘3D object counter’ plugin^40^ was then used with a threshold applied to objectively count individual foci. The same threshold was used for all images.

### Spindle morphology

For spindle morphology an automated ellipsoid-fitting algorithm (ImageJ) was applied to 3D confocal stacks (z-resolution 1 μm) to best represent the shape and size of the spindle. Then the longest two axes of the ellipsoid were reported as the spindle length and width.

### Bivalent biorientation

Analysis of bivalent biorientation was done by registering of kinetochore positions in 3D confocal stacks using in-lab ImageJ macros. Macros label kinetochores non-permanently to prevent registering of same kinetochore twice and allow the kinetochores to be registered in pairs. Subsequent macros then determined the position and normal angle of the spindle equator to best fit all the bivalents and then calculated the inter-kinetochore distance, displacement and angle of each bivalent. Oocytes matured to metaphase (NEB+8 h) in maturation media without addition of follicular fluid were used as controls. The mean and standard deviations (s.d.) for bivalent stretch, displacement and angle of bivalents in this group were used to define the standard metaphase. Then each bivalent, in the control and experimental groups was compared to this standard. The number of s.d. away from the mean was calculated for stretch, displacement and angle for each bivalent, and the worst performing metric used to color that bivalent. Colors were assigned as follows: If the worst metric is <1 s.d. from the mean is colored green; ≥1 s.d. and <2 s.d. colored yellow; ≥2 s.d. and <3 s.d. colored orange; and ≥3 s.d. colored red. So a bivalent with stretch and displacement within one s.d. of their respective control means, but with angle greater than 1 s.d. and less than 2 s.d. from the mean would be colored yellow (<2 s.d. from the mean). These are the colors used in the scatter plots in Figs 2 and S3.

All DNA damage, spindle morphology and bivalent biorientation analysis was done in a blinded fashion.

### Figure preparation

Graphs were produced in Prism and then figures assembled in Illustrator (Adobe, USA). Micrographs were transferred directly from ImageJ to Illustrator.

### Endometriosis & Follicular Fluid retrieval

All participants in the endometriosis and control groups were confirmed to have presence or absence of endometriosis respectively by laparoscopy. The severity was recorded using the ASRM classification^20^. Participants were subject to controlled ovarian stimulation, and trans-vaginal oocyte retrieval (TVOR) was performed once the conditions for oocyte retrieval were met (≥3 follicles measuring 17 mm diameter). During TVOR the follicular fluid was collected from a single follicle with a diameter >15 mm. Only FF free from blood and containing an oocyte were used in this study. FF was centrifuged at 1200 g for 20 minutes and then aliquoted into sterile tubes and frozen in liquid nitrogen before being stored at −80 °C. Samples were identified by a unique code that linked to the patient’s anonymised data. Samples were thawed for use on the same day.

## Additional Information

**How to cite this article**: Hamdan, M. *et al.* The sensitivity of the DNA damage checkpoint prevents oocyte maturation in endometriosis. *Sci. Rep.* **6**, 36994; doi: 10.1038/srep36994 (2016).

**Publisher’s note:** Springer Nature remains neutral with regard to jurisdictional claims in published maps and institutional affiliations.

## Supplementary Material

Supplementary Information
