# Supplementary material for: The sensitivity of the DNA damage checkpoint prevents oocyte maturation in endometriosis
Source: Sci Rep. 2016 Nov 14;6:36994. doi: 10.1038/srep36994 (PMC5107963; doi:10.1038/srep36994)
Supplement: Supplementary Information [file srep36994-s1.pdf]

The sensitivity of the DNA damage checkpoint prevents oocyte maturation in endometriosis.

Mukhri Hamdan[1,2], Keith T. Jones[3], Ying Cheong[1] & Simon I.R. Lane[3*]

Supplementary Figure and Table Legends

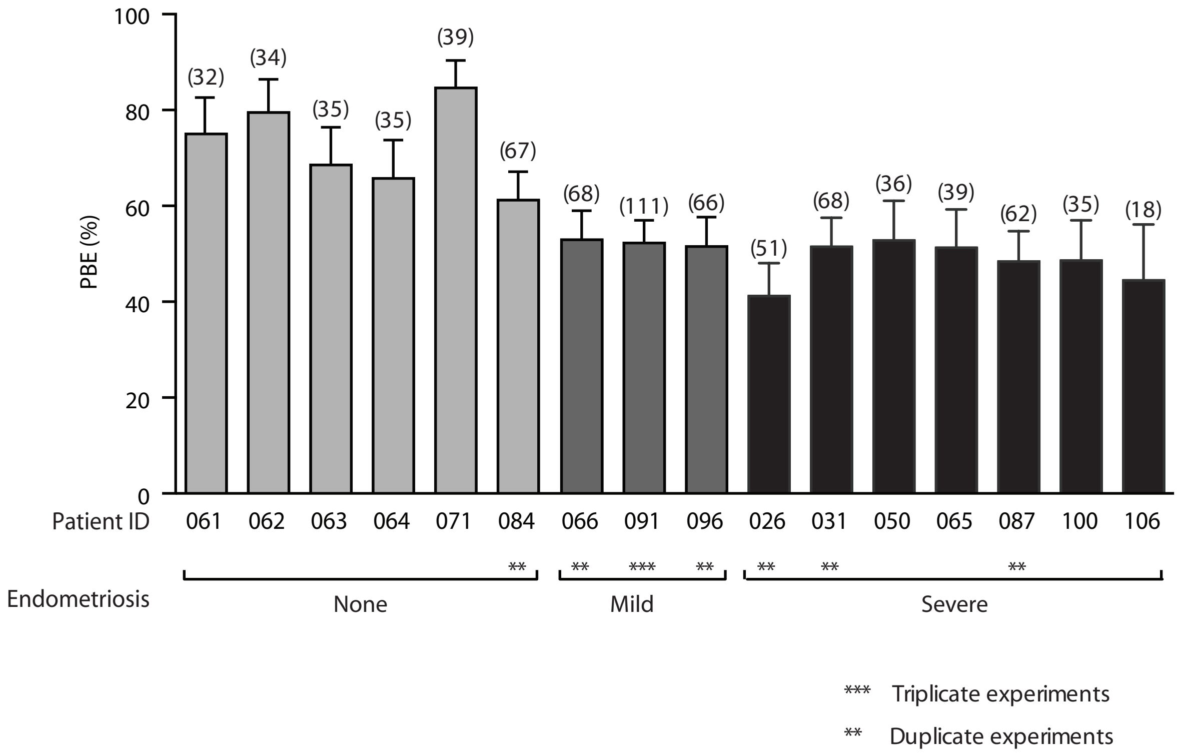

Supplementary Figure 1 Polar body extrusion rate in mouse oocytes following incubation in 50% follicular fluid from patients with no, mild or severe endometriosis. Number of oocytes are indicated in parenthesis. Error bars represent standard error.

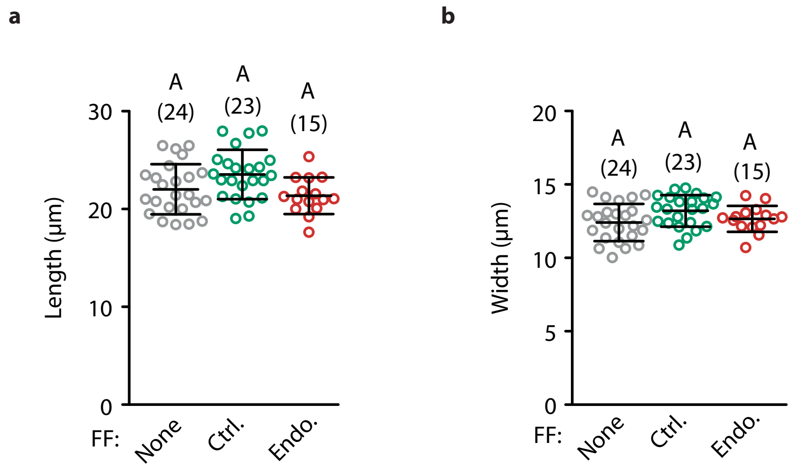

Supplementary Figure 2 Metaphase II spindle dimensions of oocytes matured in No-FF, 50% Control-FF or 50% Endo-FF , showing spindle length (**a**) and width (**b**). Number of oocytes indicated in parenthesis. Error bars represent standard deviation, (ANOVA with Tukey's post-hoc test).

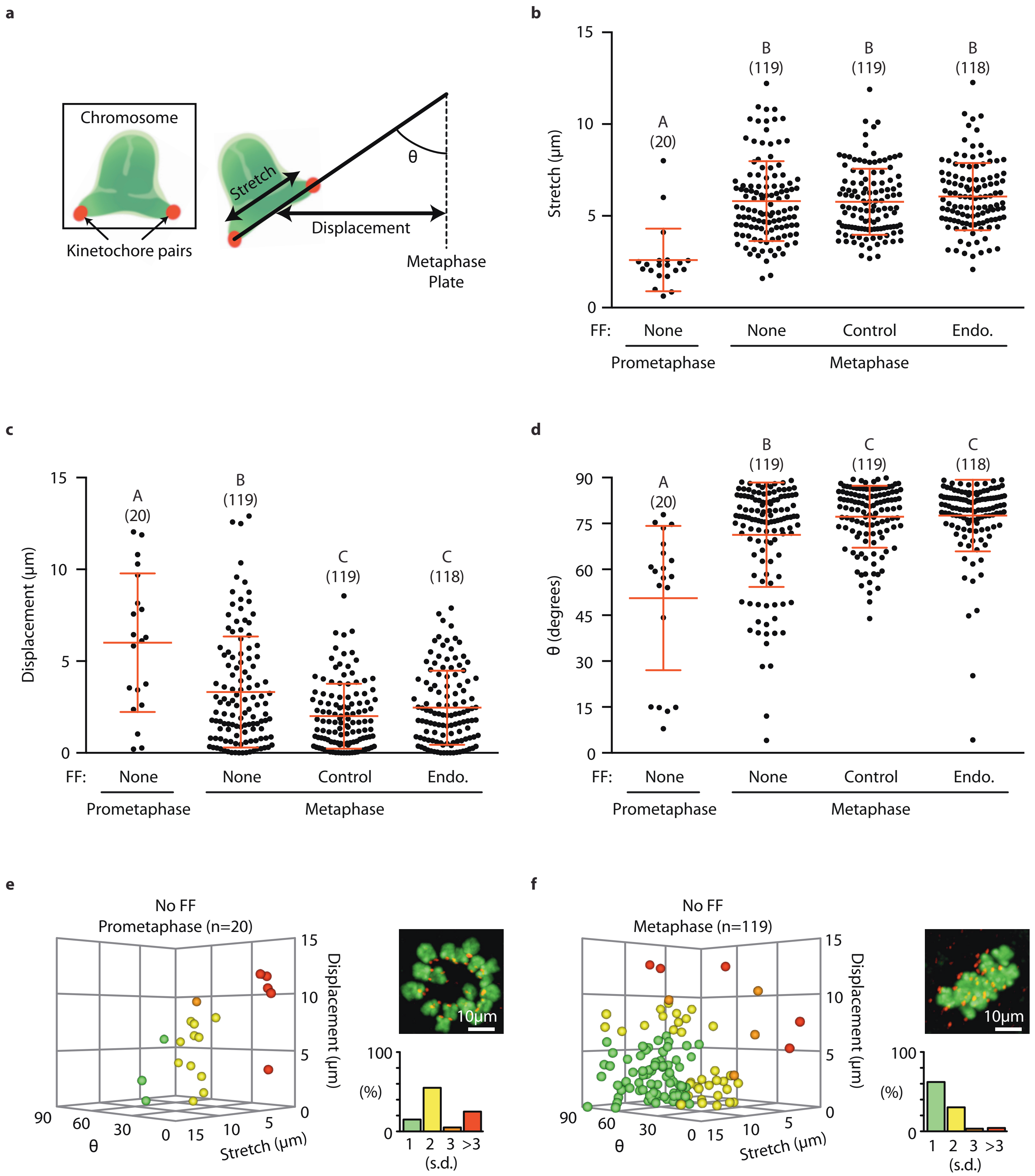

Supplementary Figure 3 Bivalent biorientation is unaffected by Endometriosis follicular fluid. (**a**) Representation of a bivalent with three measurements shown (stretch, displacement and θ) relative to the metaphase plate. (**b-d**) Bivalent stretch (b),

Displacement (c) and θ (d), as shown in (a) for bivalents in oocytes fixed at either prometaphase, or metaphase and cultured in either No-FF or Endo-FF as indicated. Red lines represent the mean and standard deviations. Number of bivalents are indicated in parenthesis. Different letters indicate significant difference ($P<0.05$, ANOVA with Tukey's post-hoc test). (**e-f**) 3D scatter plots showing bivalents from either prometaphase (e) or metaphase (f) control oocytes. Colours indicate the maximum number of standard deviations a bivalent is from the mean in any of the measures (stretch, displacement, θ) where the mean and the standard deviation are defined by the No-FF metaphase control group (green, <1s.d.; yellow <2s.d.; orange, <3s.d.; red ≥ 3 s.d.). Micrographs show representative images with centromeres (Anti-centromeric Antibody) in red and chromatin (Hoechst) in green. Bar charts show the percentage of bivalents according to the number of s.d. from the mean.

	Endometriosis (n=10)	Control (n=6)	P-value
Age (years)	33.8 ±2.7	32.8 ±2.3	0.47
BMI (kg/m^2)	24.4 ±4.1	23.5 ±2.3	0.66
Duration of infertility (Months)	42.8 ±12.6	54.0 ±34.6	0.41
Basal serum FSH	8.13 ±2.3	6.63 ±0.9	0.31
Antral Follicle Count (AFC)	11.6 ±5.1	16.0 ±6.8	0.16
Number of oocytes retrieved	8.2 ±5.6	14.7 ±5.3	0.04*

Supplementary Table 1 Summary demographic data for patients whose follicular fluid was used in the study. No statistical differences were found between endometriosis and control groups excepting the number of MII oocytes retrieved. Data are expressed as mean ± standard deviation and P values calculated by t-test.
